# Socioeconomic Macro-Level Determinants of Hypertension: Ecological Analysis of 138 Low- and Middle-Income Countries

**DOI:** 10.3390/jcdd10020057

**Published:** 2023-01-30

**Authors:** Mustapha S. Abba, Chidozie U. Nduka, Seun Anjorin, Fatima H. Zanna, Olalekan A. Uthman

**Affiliations:** 1 Division of Health Sciences, Warwick Medical School, University of Warwick, Coventry CV4 7AL, UK; 2 Deutsche Gesellschaft für Internationale Zusammenarbeit, Asokoro, Abuja 900103, Nigeria; 3 Warwick Centre for Global Health, Division of Health Sciences, Warwick Medical School, University of Warwick, Coventry CV4 7AL, UK; 4 Division of Epidemiology and Biostatistics, Department of Global Health, Stellenbosch University, Stellenbosch 7602, South Africa; 5 Department of Public Health (IHCAR), Karolinska Institutet, 171 77 Stockholm, Sweden

**Keywords:** high blood pressure, hypertension, socioeconomic determinants, government health expenditure, GDP per capita, multidimensional poverty index

## Abstract

Aim: To assess the relative importance of major socioeconomic determinants of population health on the burden of hypertension in Low-and-Middle-Income Countries (LMICs). Methods: Country-level data from 138 countries based on *World Development Indicators 2020* were used for correlation and linear regression analyses of eight socioeconomic predictors of hypertension: current health expenditure, domestic general government health expenditure per capita, GDP per capita, adult literacy rate, unemployment rate, urban population, multidimensional poverty index, and total population. Results: The median prevalence of age-standardised hypertension was 25.8% across the 138 countries, ranging from 13.7% in Peru to 33.4% in Niger. For every 10% increase in the unemployment rate, the prevalence of hypertension increased by 2.70%. For every 10% increase in the percentage of people living in urban areas, hypertension was reduced by 0.63%. Conclusions: The findings revealed that countries with high GDP, more investment in health and an improved multidimensional poverty index have a lower prevalence of hypertension.

## 1. Introduction

Hypertension, also known as high blood pressure, is a condition in which the blood vessels have persistently raised pressure in the arteries that causes the heart to work too hard [1,2]. It is considered the most important risk factor for cardiovascular disease and the most important preventable cause of mortality [3,4,5]. The prevalence of hypertension has increased significantly in the past decade, especially in low- and middle-income countries (LMICs) [6]. Between 1990 and 2020, hypertension was estimated to increase by 120% in women and 137% in men in LMICs, causing 10.4 million deaths per year in the world [1]. The highest prevalence is reported in Sub-Saharan Africa, Central Asia, and Eastern Europe [2], regions comprised mainly of LMICs [7]. High blood pressure is a preventable disease associated directly with lifestyle habits, including an unhealthy diet, physical inactivity and tobacco smoking [8]. The relationship between high blood pressure, income level, wealth, employment status and place of residence has been identified in the literature [9,10,11]. Other individual-level factors such as ethnicity, education level, and unhealthy behaviours have received much attention [12,13]. Researchers have established societal and economic factors linked to the prevalence and management of hypertension in LMICs [1,8], such as financial or human resource challenges in dealing with hypertension [14]. These determinants have a profound effect on the scale and profile of hypertension [15,16]. However, few studies were conducted to explore the association between socioeconomic determinants of population health at the country level and hypertension. Understanding the association could help improve public health and resource allocation [8,17,18].

Increased spending in the health sector, donor financing, and the redistribution of national budgets will improve the health of individuals in LMICs [19,20,21]. Recent reviews show that more investment in the health system can reduce the prevalence of hypertension [21,22]. In addition, hypertension can be lowered indirectly by improving the socioeconomic determinants of population health, influencing education, housing, gender equality, and human rights [19]. However, the importance of the underlying socioeconomic variables and the degree to which different socioeconomic factors impact hypertension is unclear [23]. Since the relative importance of improved education, health services and economic inequity may vary between countries, best strategies can be exploited within the countries undergoing an epidemiological transition from infectious to chronic diseases. Hypertension remains the most common risk factor for death [24]. Therefore, assessing the association between the prevalence of hypertension and eight major socioeconomic determinants including current health expenditure, domestic general government health expenditure per capita, GDP per capita, the adult literacy rate, unemployment, urban population, the multidimensional poverty index, and total population could provide policymakers with guidance on how to prevent hypertension at any given level of development within the context of LMICs.

## 2. Materials and Methods

### 2.1. Study Design

The study analysed the relationship between socioeconomic determinants and the prevalence of hypertension, including national-level data from 138 low- and middle-income countries as units of observation. We chose hypertension as our primary estimate of health, since data are available for almost of all the selected countries and are highly correlated to the other health outcomes.

### 2.2. Data Source

The hypertension prevalence data was sourced from World Health Organization global health observatory, which is a data repository gateway to the health-related statistics for its 194 member states. It provides access to over 1000 indicators on priority health for priority health topics, including mortality and disease burden, HIV/AIDS, TB, malaria, neglected diseases, epidemic-prone diseases, health systems, environmental health water and sanitation, NCDs and risk factors, violence and injuries, and equity, among others. Data on health expenditures, domestic general government health expenditure per capita, GDP per capita, adult literacy rate, unemployment, urban population, multidimensional poverty index and total population were extracted from the World Development Indicators 2020 database [25].

### 2.3. Outcome

The outcomes were the percentage of the defined population with increased blood pressure (systolic blood pressure ≥140 or diastolic blood pressure ≥90) calculated using age-standardised estimates according to the blood pressure. If more than one reading of a participant’s blood pressure was taken, the first reading for that participant was discarded, and the average of the remaining readings was used.

### 2.4. Socioeconomic Determinants

The following country-level socioeconomic factors included in the analysis are markers of development: current health expenditure (percentage of GDP), domestic general government health expenditure per capita, GDP per capita (in US$), adult literacy rate (for people ages 15 and above), unemployment (percentage of the total labour force), urban population (percentage of the total population), multidimensional poverty index, and total population.

### 2.5. Statistical Analyses

We performed a data analysis using the R statistical package (version 4.2.2 Vienna, Austria). Pearson correlation analyses were used to compare outcome variables and determinants. Linear regression analyses were performed to determine the association between hypertension and socioeconomic predictors, using adjusted R^2^ and 95% confidence intervals as measures of model strength. The relative importance of each variable was assessed by its partial correlation. To investigate effect modification by income level, a separate model was fitted for LMICs.

A residual analysis was used to reveal outliers and generate hypotheses for better models. Multicollinearity diagnostics were used to examine multicollinearity among the independent variables. The test included the tolerance test for multicollinearity and its reciprocal variance inflation factors (VIF). Multicollinearity is present when the VIF is higher than 10 or the mean VIF is greater than 6. The diagnosis of multi-collinearity is shown in Appendix A. The largest VIF ranged from 1.07 to 5.26, and the mean VIF was 2.51. Since none of the VIF values exceeds 10 and the mean VIF is less than 6, we concluded that there was no multi-collinearity problem [26].

## 3. Results

**Figure 1:**
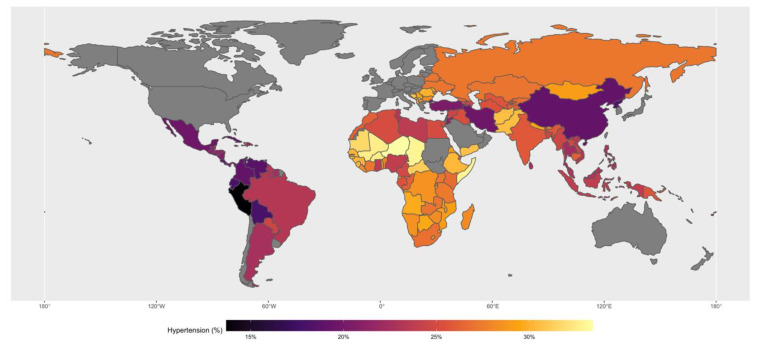
The median prevalence of age-standardised hypertension in studied countries.

A total of 138 countries from LMICs were included in this analysis. The summary characteristics of included countries are shown in Table 1. The median prevalence of age-standardised hypertension was 25.8% across the 138 countries, ranging from 13.7% in Peru to 33.4% in Niger (Figure 1). The adult literacy rate ranged from 22.3% in Chad to 99.99% in Uzbekistan. The percentage of unemployed ranged from 0.39% in Cambodia to 27.69% in Bosnia and Herzegovina. The percentage living in urban areas ranged from 12.1% in Burundi to 91.50% in Argentina.

The pair-wise correlation between hypertension and the eight factors is summarised in Table 2. There was a negative statistically significant weak correlation between hypertension prevalence and domestic healthcare expenditure (r = −0.36; 95%CI = −0.50 to −0.20), and negative statistically significant moderate correlation between hypertension, gross domestic product per capita (PPP) (r = −0.46; 95%CI = −0.59 to −0.31), adult literacy rate (r = −0.56; 95%CI = −0.67 to −0.42), and percentage living in urban areas (r = −0.46; 95%CI = −0.59 to −0.32). In addition, there was a positive statistically significant moderate correlation between hypertension prevalence and the multidimensional poverty index (r = 0.59; 95%CI = 0.45 to 0.71), indicating that the multidimensional poverty index increases with the prevalence of hypertension.

The crude and adjusted association between the country’s hypertension prevalence and socioeconomic factors is also shown in Table 2. The multivariable model accounted for 57.0% of the variation in countries’ hypertension prevalence estimates. In the adjusted analyses, where all of the eight factors were controlled, only the unemployment rate, the percentage living in an urban area, and the multidimensional poverty index were statistically significantly associated with hypertension prevalence. As the country’s multidimensional poverty index increases, the country’s hypertension prevalence increases by 0.06%. On the other hand, for every 10% increase in the unemployment rate, the prevalence of hypertension increased by 2.70%. For every 10% increase in the percentage of people living in urban areas, hypertension reduced by 0.63%.

## 4. Discussion

This ecological study found some evidence of variation between countries. We found that countries with high GDP, more investment in health and an improved multidimensional poverty index have a lower prevalence of hypertension. Even though it is increasingly acknowledged that the burden of hypertension in low- and middle-income nations is a public health concern, population-based studies and their determinants remain scarce in the most affected region of the world [27,28]. Much study has focused on socio-demographic characteristics at the individual level. However, theories show that the distribution and determinants of population health are multilayered from an epistemic standpoint. Utilizing a socio-ecological model, we investigated macro-level socioeconomic determinants linked with hypertension. The socio-ecological approach conceptualises the hypertension burden as a multidimensional phenomenon rooted in the interaction between individual, community, and societal influences. The framework takes into account the various organisational levels of society and their influence on the risk of hypertension. The results demonstrated an association between GDP, domestic health expenditure, and the prevalence of hypertension. Both determinants are common macroeconomic indicators used to measure the standard of living and public health policies [29]. The study also revealed that countries with high GDP and domestic health expenditure had a lower prevalence of hypertension. This finding is consistent with the study of Gheorghe and colleagues [18]. The burden of cardiovascular diseases was higher in lower-income countries where health expenditures were smaller in the absolute. Concurrently, cardiovascular diseases cause significant reductions in GDP, suggesting a reciprocal relationship [18]. These findings highlight the need for more investment in the health sector to manage the burden of hypertension across limited-resource settings.

The study found a positive association between a country’s multidimensional poverty index, unemployment rate, and hypertension prevalence. Increased multidimensional poverty indices and unemployment rates increased the country’s hypertension prevalence. There are several possible explanations for the association between a multidimensional poverty index and a high hypertension prevalence. For example, cardiovascular risk factors are more prevalent, and access to medical care is more limited in developing countries [22], in addition to the yearly increasing hypertension care cost [22]. Another explanation could be that poorer countries allocate a lower proportion of their economic resources to health when compared to wealthier countries [29]. Inefficient health systems and inadequate government expenditures on health promotion interventions may also explain the high prevalence of hypertension [30,31]. Many of these countries, for example, still lack access to and are unable to afford CVD secondary preventive medications. Individuals, households, financial agents, public institutions, government, and society suffer economic repercussions because of CVD-related disability. Not only is this burden expected to expand in the future, but LMICs are also expected to bear a more significant portion of it because of population growth, ageing, and globalisation. In LMICs, the economic burden of CVD on households, health systems, and national incomes could threaten current poverty-reduction efforts. The societal cost of hypertension is also significant, as patients with lower productivity or who are unable to work owing to illness would be financially impacted and their households’ finances remain unstable (19). The study found a negative correlation between hypertension prevalence and the adult literacy rate. People in countries with high literacy rates are likely to be aware of their hypertension status and seek medical treatment compared to countries with a low literacy rate. This leads to reduced hypertension prevalence as literacy increases. In addition, early consultation and access to healthcare were associated with hypertension awareness and management [32].

The study showed that residence areas were associated with the prevalence of hypertension, with less hypertension prevalence among people living in urban areas compared to people from rural areas. It is important to note that city dwellers have a greater level of education and a higher rate of literacy than the general population; as a result, their living standards are higher, which can contribute to improved health behaviours among this demographic and reduce the prevalence of hypertension.

Though an increase in population is a global challenge being confronted by LMICs, our findings showed that the prevalence of hypertension reduces by 0.63% for each 10% increase in the percentage of people living in urban areas. When managing rapid urbanisation, urban planning reduces non-communicable diseases by encouraging walking, cycling and public transport. In this regard, creating safer, attractive neighbourhoods and affordable means of transportation enable healthier and more sustainable environmental, social and behavioural choices for compact city residents [33]. This, together with the general improvements in overall hypertension care for those with low economic status, could perhaps explain the reduction in hypertension prevalence. Using antihypertensive medication was linked to having access to public health facilities. Primary healthcare has been steadily improving, which appears to have contributed to increasing antihypertensive drug use, resulting in better blood pressure control [32].

### 4.1. Limitations

The study included a large data set across 138 countries. There are some limitations to be considered when interpreting the findings of this study. The data collection strategy was standardised. However, the reporting system in each country might differ. Secondly, data for some countries might be missing, or some countries have no data; hence the findings should be generalised with caution. Ecological studies provide a quick and easy way of determining associations between factors of interest and outcome but the inability to characterise potential cofounders makes it challenging to draw definitive conclusions and hence cannot determine causality [34]. The current ecological study adds to the literature demonstrating the importance of socioeconomic determinants and hypertension in LMICs. The findings suggest that countries with a pronounced burden of hypertension also have increased poverty, high employment, and invest less to health, affecting the economy and health systems. However, they highlight the need for further research to understand how socioeconomic determinants interact in such populations at individual levels.

Further research is needed to identify innovative ways of integrating hypertension prevention and care strategies in different populations in LMICs and the cost-effective ways of implementing policies to address the high prevalence of hypertension.

### 4.2. Policy Implications

Health expenditure is increasing substantially relative to GDP growth in almost all countries at all income levels. This increase in expenditure has become a significant concern for governments and policymakers. The health sector should lead in advocacy for integrated multi-sector urban planning that prioritises health sustainability, particularly in rapid changing LMICs. Like the HICs, LMICs should base their economic growth on creating and using knowledge. LMICs should increase the gross economic expenditure on research and development to increase understanding to deploy new technology to reduce the burgeoning epidemic of hypertension [17]. It is important to involve patients in the clinical decision-making process and access to multi-disciplinary care [35].

Socioeconomic determinants are an essential benchmark of health system performance for managing hypertension in LMICs against which future progress can be compared, implying that lessons could be learned from approaches adopted by those health systems. Assessing the health system’s success in managing important, yet inexpensive, treatable non-communicable disease risk factors, including hypertension, would be a valuable measure of health system performance that could feasibly be tracked as part of national and international targets, such as moving toward universal health coverage. Specifically, as LMICs undergo the epidemiological transition from infectious to chronic diseases, such health system performance measures could help track countries’ progress in shifting health services to chronic condition care [6]. Standardising healthcare costs across countries for ease of reference and comparisons could enable better monitoring and facilitate decisions involving fund allocation.

## 5. Conclusions

The findings revealed that countries with high GDP, more investment in health and an improved multidimensional poverty index have a lower prevalence of hypertension.

## Figures and Tables

**Table 1:** Summary characteristics of variables included in the analyses.

Country	Raised Blood Pressure (SBP ≥ 140 OR DBP ≥ 90), Age-Standardized (%)	Current Health Expenditure (% of GDP)	Domestic General GovernMent Health Expenditure Per Capita	GDP Per Capita (Current US$)	Literacy Rate, Adult Total (% of People Ages 15 and Above)	Unemployment, Total (% of the Total Labour Force)	Urban Population (% of the Total Population)	Multidimensional Poverty Index	Total Population
Afghanistan	30.6	10.1	10.9	556.0	31.4	11.1	24.8	55.9	34,413,603
Angola	29.7	2.6	90.6	4167.0	66.0	7.4	63.4	51.1	27,884,380
Albania	29	4.9	317.4	3952.8	98.1	17.2	57.4	0.7	2,880,703
Argentina	22.6	10.2	1373.9	13,789.1	99.0	7.5	91.5	N/A	43,131,966
Armenia	25.5	10.1	160.5	3607.3	99.8	18.3	63.1	0.2	2,925,559
Azerbaijan	24.5	4.1	195.1	5500.3	99.8	5.0	54.7	N/A	9,649,341
Burundi	29.2	6.4	20.4	305.5	68.4	1.6	12.1	74.3	10,160,034
Benin	27.7	2.9	16.9	1076.8	42.4	2.0	45.7	66.8	10,575,962
Burkina Faso	32.6	5.1	25.0	653.3	39.3	4.3	27.5	83.8	18,110,616
Bangladesh	24.7	2.6	16.5	1248.5	74.9	4.4	34.3	24.6	156,256,287
Bulgaria	28.4	7.4	757.3	7074.7	98.4	9.1	74.0	N/A	7,177,991
Bosnia and Herzegovina	30.8	9.4	777.4	4729.7	97.0	27.7	47.2	2.2	3,429,362
Belarus	27.1	6.1	668.7	5967.1	99.9	5.8	77.2	N/A	9,461,076
Belize	22.7	5.9	292.3	4770.2	N/A	7.6	45.4	4.3	360,926
Bolivia	17.9	6.6	307.2	3036.0	92.5	3.1	68.4	20.4	10,869,732
Brazil	23.3	8.9	568.2	8814.0	93.2	8.4	85.8	3.8	204,471,759
Bhutan	28.1	3.8	252.6	2752.6	66.6	2.5	38.7	37.3	727,885
Botswana	29.6	5.7	603.2	6402.9	86.8	20.6	67.2	17.2	2,120,716
Central African Republic	31.2	5.0	3.6	377.4	37.4	5.6	40.3	79.4	4,493,171
China	19.2	4.9	377.7	8016.4	96.8	4.6	55.5	3.9	1,379,860,000
Cote d’ Ivoire	27.2	3.2	37.4	1972.5	89.9	3.1	49.4	46.1	23,226,148
Cameroon	24.8	3.7	12.4	1382.5	77.1	3.6	54.6	45.3	23,298,376
Congo, Dem. Rep.	28.5	4.0	6.0	497.3	77.0	4.5	42.7	64.5	76,244,532
Congo, Rep.	26.2	2.5	51.9	2447.5	80.3	20.4	65.5	24.3	4,856,093
Colombia	19.2	7.5	699.9	6175.9	95.6	8.3	79.8	4.8	47,520,667
Comoros	27.9	4.6	12.6	1242.6	58.8	8.1	28.5	37.3	777,435
Cabo Verde	29.5	4.8	190.3	3043.0	86.8	11.8	64.3	N/A	524,740
Costa Rica	18.7	7.6	943.4	11,642.8	97.9	9.0	76.9	N/A	4,847,805
Cuba	19	12.8	2872.8	7694.0	99.8	2.4	76.9	0.4	11,324,777
Djibouti	26.8	3.1	77.9	2658.9	N/A	26.3	77.4	N/A	913,998
Dominica	22.5	5.2	362.4	7597.3	N/A	N/A	69.6	N/A	71,175
Dominican Republic	21.5	5.8	349.1	6921.5	93.8	7.6	78.6	3.9	10,281,675
Algeria	25.1	7.0	590.8	4177.9	81.4	11.2	70.8	2.1	39,728,020
Ecuador	17.9	7.5	477.0	6124.5	93.6	3.6	63.4	4.6	16,212,022
Egypt, Arab Rep.	25	5.3	191.9	3562.9	71.2	13.1	42.8	5.2	92,442,549
Eritrea	29.1	4.5	11.8	N/A	76.6	5.8	N/A	N/A	N/A
Ethiopia	30.3	3.8	16.0	640.5	51.8	2.3	19.4	83.5	100,835,453
Fiji	21.7	3.3	260.5	5390.7	N/A	4.3	54.7	N/A	868,632
Micronesia, Fed. Sts.	25	12.5	94.7	2906.6	N/A	N/A	22.5	N/A	108,886
Gabon	25.5	2.7	230.1	7384.7	84.7	20.6	88.1	14.8	1,947,690
Georgia	26.3	7.4	295.5	4014.2	99.6	16.5	57.4	0.3	3,725,276
Ghana	23.7	4.6	83.0	1774.1	79.0	6.8	54.1	30.1	27,849,203
Guinea	30.3	5.8	7.8	769.3	39.6	4.9	35.1	66.2	11,432,096
Gambia, The	29.1	3.2	20.6	660.7	50.8	9.5	59.2	41.6	2,085,860
Guinea-Bissau	30.3	8.1	9.4	603.4	45.6	5.9	42.1	67.3	1,737,207
Equatorial Guinea	28.4	2.9	133.3	11,283.4	94.4	8.5	70.6	N/A	1,168,575
Grenada	24.3	4.6	273.8	9096.5	98.6	N/A	36.0	N/A	109,603
Guatemala	21.2	6.0	178.8	3994.6	80.8	2.5	50.0	28.9	15,567,419
Guyana	23.1	4.0	263.9	5576.8	85.6	13.2	26.4	3.4	767,433
Honduras	21.4	7.5	142.2	2286.2	88.5	6.2	55.2	19.3	9,112,904
Haiti	24.5	5.1	16.3	1386.9	61.7	14.0	52.4	41.3	10,695,540
Indonesia	23.8	2.9	118.7	3331.7	96.0	4.5	53.3	3.6	258,383,257
India	25.8	3.6	50.4	1605.6	74.4	5.4	32.8	27.9	1,310,152,392
Iran, Islamic Rep.	19.7	7.5	531.5	4904.3	85.5	11.2	73.4	N/A	78,492,208
Iraq	25.2	3.1	75.9	4688.3	85.6	10.7	69.9	8.6	35,572,269
Jamaica	21.8	5.6	312.9	4907.9	88.1	13.5	54.8	4.7	2,891,024
Jordan	21	7.5	378.1	4164.1	98.2	13.1	90.3	0.4	9,266,573
Kazakhstan	27.1	3.0	445.1	10,510.8	99.8	4.9	57.2	0.5	17,542,806
Kenya	26.7	5.2	70.9	1464.6	81.5	2.8	25.7	38.7	47,878,339
Kyrgyz Republic	26.7	7.1	114.2	1121.1	99.6	7.6	35.8	0.4	5,956,900
Cambodia	26.1	6.2	45.6	1162.9	80.5	0.4	22.2	37.2	15,521,435
Kiribati	21.5	8.0	143.7	1542.6	N/A	N/A	51.6	19.8	110,927
Lao PDR	24.8	2.5	53.3	2140.0	84.7	0.8	33.1	23.1	6,741,160
Lebanon	20.7	7.4	500.3	7663.9	95.1	9.3	88.1	N/A	6,532,681
Liberia	28.3	10.6	15.2	721.6	48.3	2.1	49.8	62.9	4,472,229
Libya	23.7	N/A	N/A	4337.9	N/A	19.5	79.3	2.0	6,418,315
St. Lucia	27.1	4.6	269.9	10,093.6	N/A	20.6	18.5	1.9	179,131
Sri Lanka	22.4	3.9	198.2	3843.8	92.3	4.5	18.3	2.9	20,970,000
Lesotho	29	9.0	165.2	1146.1	76.6	23.8	26.9	19.6	2,059,011
Morocco	26.1	5.1	150.1	2875.3	73.8	9.5	60.8	18.6	34,663,608
Moldova	29.8	8.6	287.6	2732.5	99.4	4.7	42.5	0.9	2,834,530
Madagascar	28.1	5.0	30.8	467.2	76.7	1.8	35.2	69.1	24,234,080
Maldives	24.4	8.7	1050.7	9033.4	97.7	6.9	38.5	0.8	454,914
Mexico	19.7	5.7	546.7	9616.6	95.2	4.3	79.3	6.6	121,858,251
Marshall Islands	21.3	17.0	195.4	3199.9	98.3	N/A	75.8	N/A	57,444
North Macedonia	28.5	6.3	569.6	4861.6	98.4	26.1	57.4	2.5	2,070,226
Mali	32.6	4.1	18.7	751.5	30.8	7.7	40.0	68.3	17,438,772
Myanmar	24.6	5.5	49.5	1196.7	89.1	0.8	29.9	38.3	52,680,724
Montenegro	29.1	9.0	874.6	6514.3	98.8	17.5	65.8	1.2	622,159
Mongolia	29	4.2	257.3	3875.3	99.2	4.9	68.2	7.3	2,998,433
Mozambique	29.1	6.7	23.4	589.9	60.7	3.4	34.4	72.5	27,042,001
Mauritania	31.7	3.7	59.6	1524.1	53.5	10.1	51.1	50.6	4,046,304
Mauritius	25	5.7	457.9	9260.4	91.3	7.4	41.0	N/A	1,262,605
Malawi	28.9	9.3	27.4	380.6	62.1	5.9	16.3	52.6	16,745,305
Malaysia	22.9	3.8	504.4	9955.2	95.0	3.1	74.2	N/A	30,270,965
Namibia	28.5	10.0	447.9	4896.6	91.5	20.9	46.9	38.0	2,314,901
Niger	33.4	5.3	12.7	484.2	35.0	0.5	16.2	90.5	20,001,663
Nigeria	23.9	3.6	32.0	2687.5	62.0	4.3	47.8	46.4	181,137,454
Nicaragua	20.8	8.0	236.4	2049.9	82.6	4.7	57.9	16.3	6,223,234
Nepal	29.4	5.5	27.2	901.7	67.9	3.1	18.6	34.0	27,015,033
Pakistan	30.5	2.7	32.4	1356.7	58.0	3.6	36.0	38.3	199,426,953
Panama	19.9	6.8	1089.2	13,630.3	95.7	3.0	66.7	N/A	3,968,490
Peru	13.7	5.0	353.7	6229.1	94.5	3.3	77.4	7.4	30,470,739
Philippines	22.6	3.9	105.5	3001.0	96.3	3.1	46.3	5.8	102,113,206
Papua New Guinea	25.6	1.8	54.7	2679.3	61.6	2.5	13.0	56.6	8,107,772
Korea, Dem. People’s Rep.	18.2	N/A	N/A	N/A	N/A	2.7	61.3	N/A	25,183,832
Paraguay	24.6	6.7	362.6	5413.8	94.5	4.6	60.8	4.5	6,688,746
Romania	30	4.9	830.8	8969.1	98.8	6.8	53.9	N/A	19,815,616
Russian Federation	27.2	5.3	756.3	9313.0	99.7	5.6	74.1	N/A	144,096,870
Rwanda	26.7	6.6	38.0	751.1	73.2	1.1	17.0	54.4	11,369,066
Senegal	30.2	4.4	32.0	1219.2	51.9	6.8	45.9	53.2	14,578,450
Solomon Islands	22	4.6	78.5	2167.1	N/A	0.7	22.4	N/A	603,133
Sierra Leone	30.3	20.4	26.2	588.2	43.2	4.7	40.8	57.9	7,171,909
El Salvador	18.7	7.6	372.8	3705.6	89.1	4.0	69.7	7.9	6,325,121
Somalia	32.9	N/A	N/A	386.4	N/A	18.9	43.2	N/A	13,797,204
Serbia	29.5	8.8	756.9	5589.0	99.5	17.7	55.7	0.3	7,095,383
Sao Tome and Principe	25.8	5.3	64.8	1584.8	92.8	13.8	70.2	22.1	199,439
Suriname	22.4	6.2	544.6	9168.2	94.4	7.2	66.1	2.9	559,136
Eswatini	29.8	7.1	259.8	3680.3	88.4	23.3	23.3	19.2	1,104,038
Syrian Arab Republic	24.5	N/A	N/A	916.4	N/A	8.7	52.2	7.4	17,997,411
Chad	32.9	4.5	17.5	776.0	22.3	1.1	22.5	85.7	14,110,971
Togo	28.9	5.0	17.8	570.9	66.5	2.2	40.1	37.6	7,323,162
Thailand	22.3	3.7	434.9	5840.1	93.8	0.6	47.7	0.8	68,714,519
Tajikistan	26.1	6.9	61.2	978.4	99.8	7.6	26.7	7.4	8,454,019
Turkmenistan	25.4	6.3	206.5	6432.7	99.7	4.1	50.3	0.4	5,565,283
Timor-Leste	27.6	7.7	128.3	1332.8	68.1	4.4	29.5	45.8	1,196,294
Tonga	23.7	4.7	159.8	4336.2	99.4	2.5	23.3	N/A	100,780
Tunisia	23.2	6.6	365.0	4094.8	79.0	15.2	68.1	0.8	11,179,951
Turkey	20.3	4.1	828.4	11,006.3	96.7	10.2	73.6	N/A	78,529,413
Tuvalu	23.7	16.7	473.9	3197.8	N/A	N/A	59.7	N/A	11,099
Tanzania	27.3	3.6	28.7	947.9	77.9	2.1	31.6	55.4	51,482,638
Uganda	27.3	5.1	17.2	847.3	76.5	1.9	22.1	55.1	38,225,447
Ukraine	27.1	7.8	356.9	2124.7	100.0	9.1	69.1	0.2	45,154,036
Uzbekistan	25.6	5.0	159.4	2754.0	100.0	5.2	50.8	N/A	31,298,900
St. Vincent and the Grenadines	23.3	4.1	306.2	6921.7	N/A	19.1	51.0	N/A	109,135
Venezuela, RB	18.6	4.3	337.1	N/A	97.1	6.1	88.2	N/A	30,081,827
Vietnam	23.4	4.6	144.2	2085.1	95.8	1.9	33.8	4.9	92,677,082
Vanuatu	24.2	4.3	59.6	2695.7	87.5	1.9	25.0	N/A	271,128
Samoa	24	5.8	266.8	4071.9	99.1	8.5	18.9	N/A	193,510
Yemen, Rep.	30.7	4.3	11.2	1601.8	N/A	13.8	34.8	47.7	26,497,881
South Africa	26.9	8.8	629.8	6259.8	95.0	25.1	64.8	6.3	55,386,369
Zambia	27.1	4.4	71.7	1338.3	86.7	10.1	41.9	47.9	15,879,370
Zimbabwe	28.2	7.5	41.6	1445.1	88.7	4.8	32.4	25.8	13,814,642
Sudan	N/A	7.3	100.4	1329.6	60.7	17.5	33.9	52.3	38,902,948
South Sudan	N/A	N/A	N/A	1119.7	34.5	12.3	18.9	91.9	10,715,657
American Samoa	N/A	N/A	N/A	12,059.6	N/A	N/A	87.2	N/A	55,806
Kosovo	N/A	N/A	N/A	3520.8	N/A	N/A	N/A	N/A	1,788,196
West Bank and Gaza	N/A	N/A	N/A	3272.2	97.5	23.0	75.4	1.0	4,270,092

N/A: Not Available.

**Table 2:** Country-level factors associated with prevalence estimates across countries in LMICs.

	Pearson Correlation	Unadjusted Association	Adjusted Association
Current health expenditure (% of GDP)	−0.07 (−0.24 to 0.10)	−0.93 (−3.26 to 1.40)	−0.03 (−2.25 to 2.19)
Domestic general government health expenditure per capita, PPP (in US$)	−0.36 (−0.50 to −0.20)	−0.04 (−0.06 to −0.02)	0.00 (−0.02 to 0.02)
GDP per capita (in US$)	−0.46 (−0.59 to −0.31)	−0.55 (−0.73 to −0.36)	−0.08 (−0.44 to 0.28)
Literacy rate, adult total (% of people ages 15 and above)	−0.56 (−0.67 to −0.42)	−1.12 (−1.43 to −0.82)	−0.37 (−0.88 to 0.14)
Unemployment, total (% of total labour force)	0.18 (0.00 to 0.34)	1.06 (0.01 to 2.10)	2.70 (1.82 to 3.58)
Urban population (% of total population)	−0.46 (−0.59 to −0.32)	−0.89 (−1.19 to −0.59)	−0.63 (−1.00 to −0.26)
Multidimensional poverty index	0.59 (0.45 to 0.71)	0.09 (0.06 to 0.11)	0.06 (0.01 to 0.10)
Total population	−0.12 (−0.29 to 0.05)	−0.03 (−0.07 to 0.01)	−0.02 (−0.04 to 0.01)

PPP: GDP per capita; GPD: Gross Domestic Product; US$: United States Dollars.

## Data Availability

The data used in the study is freely available in the public domain (https://www.worldbank.org/en/about/legal/terms-of-use-for-datasets (accessed on 1 December 2022) and does not contain any personal identifying information. No ethical approval was required for the use of the data.

## Appendix A

**Table A1:** Multicollinearity Diagnostics Results.

Variable	VIF	SQRT VIF	Tolerance	R-Squared
CHE	1.33	1.15	0.754	0.246
DHE	2.23	1.49	0.4487	0.5513
GDP	3.15	1.77	0.3178	0.6822
Literacy	3.97	1.99	0.2519	0.7481
Unemployment	1.27	1.13	0.7888	0.2112
Urban	1.82	1.35	0.5492	0.4508
MPI_person	5.26	2.29	0.19	0.81
Population	1.07	1.04	0.9305	0.0695
Mean VIF	2.51			
